# ﻿Discovery of a new cryptic *Achalinus* Peters, 1869 (Serpentes, Xenodermidae) species from Hunan Province, China

**DOI:** 10.3897/zookeys.1181.109462

**Published:** 2023-09-28

**Authors:** Shun Ma, Yu-Hao Xu, Shuo Qi, Ying-Yong Wang, Shan-Shan Tang, Song Huang, Jian-Ping Jiang

**Affiliations:** 1 Chengdu Institute of Biology, Chinese Academy of Sciences, Chengdu 610041, China Chengdu Institute of Biology, Chinese Academy of Sciences Chengdu China; 2 University of Chinese Academy of Science, Beijing 100049, China University of Chinese Academy of Science Beijing China; 3 School of Life Sciences, Anhui Agricultural University, Hefei 230036, China Anhui Agricultural University Hefei China; 4 Anhui Province Key Laboratory of the Conservation and Exploitation of Biological Resource, College of Life Sciences, Anhui Normal University, Wuhu 241000, China Anhui Normal University Wuhu China; 5 State Key Laboratory of Biocontrol/The Museum of Biology, School of Life Sciences, Sun Yat-sen University, Guangzhou 510275, China Sun Yat-sen University Guangzhou China; 6 Hunan Institute of Agricultural Information and Engineering, Changsha 410125, China Hunan Institute of Agricultural Information and Engineering Changsha China

**Keywords:** Identification key, molecular systematics, morphological characters, snake, taxonomy

## Abstract

A new species, *Achalinussheni***sp. nov.**, from central Hunan Province is described, based on the results of molecular systematics and morphological characters according to five specimens. Our molecular phylogeny inferred from the mitochondrial *CO1* gene fragment revealed that this new species is most closely related to *A.yunkaiensis*, but a considerable amount of genetic divergence exists between them (*p*-distance ranging from 5.8% to 6.4%) and much distinct genetic divergence exists compared with other known *Achalinus* species (*p*-distance ranging from 10.4% to 15.8%), supporting its validity. Morphologically, it can be distinguished from its congeners by: (1) dorsal scales strongly keeled, 23 rows throughout the body, the outmost row smooth and significantly enlarged; (2) tail relatively short, TaL/TL 0.183 ~ 0.224; (3) the suture between internasals subequal to the suture between prefrontals; (4) loreal one, subrectangular, LorH/LorL 0.53 ~ 0.57; (5) ventrals 161–170, anal entire, subcaudals 55–61, not paired; (6) the length of supraocular equal to or longer than the length of upper anterior temporal; and (7) vertebral line inconspicuous and subcaudal streak absent. Currently, 27 species of *Achalinus* are known in the world, amongst which 20 species are distributed in China. Moreover, a key to species of the genus *Achalinus* is provided in this study.

## ﻿Introduction

The odd-scaled snakes (burrowing snakes), genus *Achalinus* Peters, 1869, are a group of small to medium-sized, nocturnal, fossorial, low-aggressive and non-venomous snakes, widely distributed in Vietnam, China and Japan (Zhao et al. 1998; Zhao 2006). Currently, 26 species of this genus have been documented and more than half of them (17 species) were described in the past five years (Wang et al. 2019; Ziegler et al. 2019; Li et al. 2020; Luu et al. 2020; Miller et al. 2020; Hou et al. 2021; Huang et al. 2021; Li et al. 2021; Ha et al. 2022; Yang et al. 2022; Ma et al. 2023b; Pham et al. 2023; Yang et al. 2023). Although a great deal of taxonomic studies have been conducted recently, the interspecific and intraspecific relationships of *A.ater* Bourret, 1937, *A.formosanus* Boulenger, 1908, *A.huangjietangi* Huang, Peng & Huang, 2021, *A.niger* Maki, 1931, *A.rufescens* Boulenger, 1888 and *A.spinalis* Peters, 1869 remain unresolved (Zhao et al. 1998; Miller et al. 2020; Huang et al. 2021; Ma et al. 2023a, 2023b; Zhang et al. 2023). Additionally, there is also a lack of molecular information for *A.hainanus* Huang, 1975 and *A.werneri* Van Denburgh, 1912, indicating that the phylogenetic positions of these species are unknown. Therefore, it is important to continue conducting relevant research of this diversity-underestimated and poorly-known genus.

**Figure 1.**
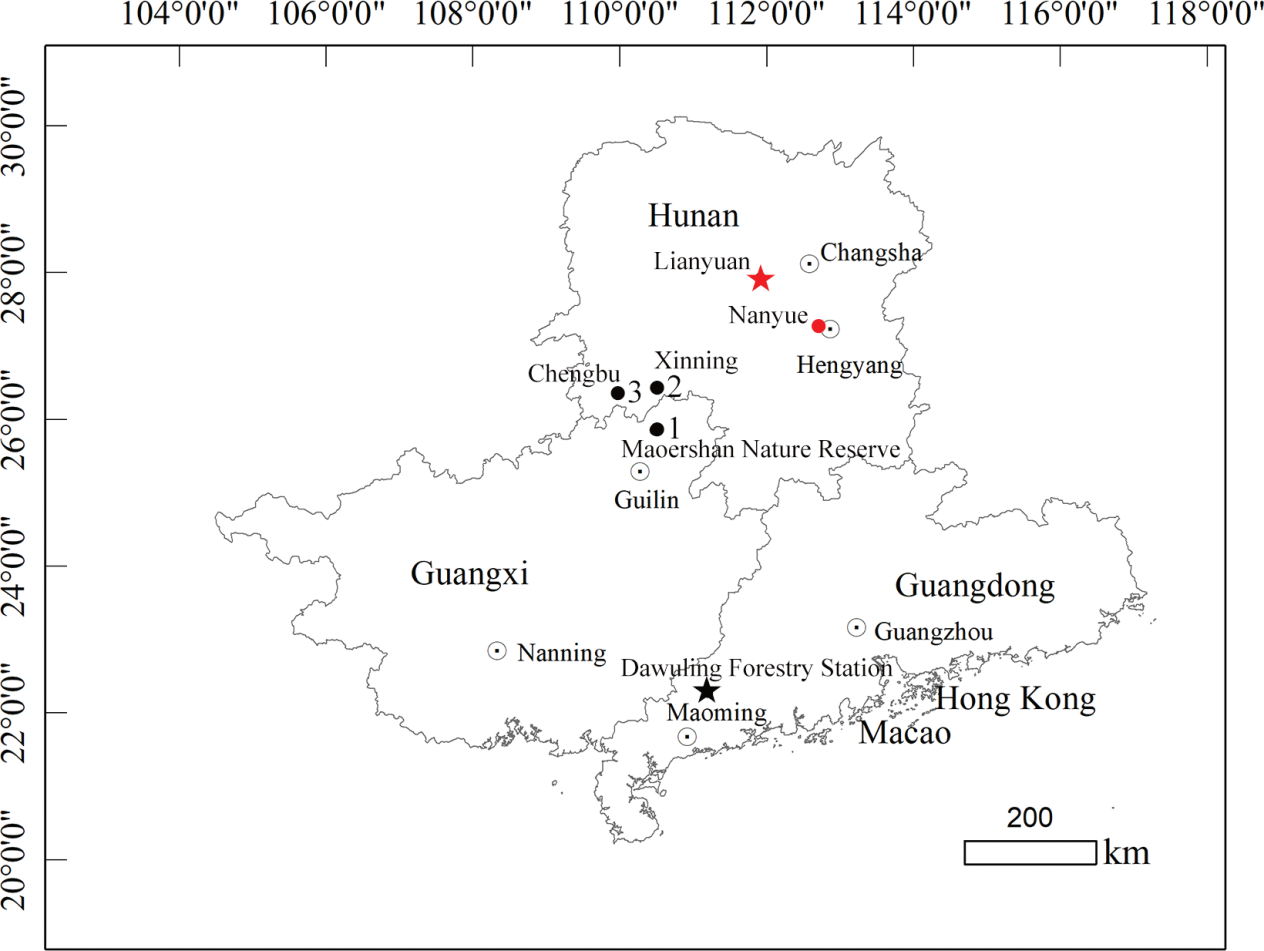
Distribution of *Achalinussheni* sp. nov. and *A.yunkaiensis*. *A.sheni* sp. nov.: the type locality (red star) and another site (red dot). *A.yunkaiensis*: the type locality (black star) and other three sites (black dots).

During our recent herpetological field survey in Hunan Province, China, five snakes were collected (Fig. 1). These specimens were assigned to *Achalinus* by their small, slender and cylindrical body shapes; lanceolate-shaped, metallic lustre and strongly-keeled dorsal scales; and absence of the preocular and postocular. However, they could not be identified as any particular *Achalinus* species morphologically. Furthermore, preliminary molecular analyses supported that these specimens comprise a separate evolutionary lineage; thus, we described them as a new species through further data analysis and investigation herein. Moreover, a key to species of the genus *Achalinus* is provided in this study.

## ﻿Materials and methods

### ﻿Molecular phylogenetic analyses

Five specimens were collected in the Hunan Province of China: four specimens (ANU20230012–ANU20230015) were collected in Lianyuan City and one specimen (CIB 119043) was collected in the Nanyue District (Fig. 1). Snakes were humanely euthanised with an injection of 0.7% tricaine methanesulphonate (MS222) solution and fresh liver tissue was extracted and immediately preserved in 95% ethanol. The specimens were fixed in 10% formalin for one day, subsequently preserved in 75% ethanol and deposited in the Anhui Normal University Museum (**ANU**) and Chengdu Institute of Biology (**CIB**) of Chinese Academy of Sciences (**CAS**), respectively. Sampling procedures involving live snakes were in accordance with the Wild Animals Protection Law of China.

Genomic DNA was extracted from the preserved liver tissues using QIAamp DNA Mini Kit (QIAGEN, Changsheng Biotechnology Co. Ltd.). A fragment of the mitochondrial cytochrome c oxidase subunit 1 (*CO1*) gene was amplified using the primer pairs: dglco and dghco (Meyer et al. 2005). The polymerase chain reaction (PCR) was performed in 25 μl reactant with the following cycling conditions: 95 °C for 4 min; 35 cycles of denaturing at 95 °C for 30 s, annealing at 48 °C for 30 s and extending at 72 °C for 60 s; and a final extending step of 72 °C for 10 min (Wang et al. 2019). PCR products were sequenced by Beijing Qingke New Industry Biotechnology Co., Ltd.

For our phylogenetic analysis, 38 sequences were used (Table 1), amongst which 33 (No. 6–38) were obtained from GenBank including 30 sequences of 23 *Achalinus* species and three sequences of *Fimbriosklossi* Smith, 1921, *Parafimbrioslao* Teynié, David, Lottier, Le, Vidal & Nguyen, 2015 and *Xenodermusjavanicus* Reinhardt, 1836, which were used as outgroups (Ma et al. 2023b).

**Table 1.** Localities, voucher information, GenBank numbers and references for all samples used in this study.

NO.	Species name	Locality	Voucher NO.	GenBank No.	References
1	*A.sheni* sp. nov.	Lianyuan, Hunan, China	ANU20230012	OR178145	This study
2	*A.sheni* sp. nov.	Lianyuan, Hunan, China	ANU20230013	OR178146	This study
3	*A.sheni* sp. nov.	Lianyuan, Hunan, China	ANU20230014	OR178147	This study
4	*A.sheni* sp. nov.	Lianyuan, Hunan, China	ANU20230015	OR178148	This study
5	*A.sheni* sp. nov.	Nanyue, Hunan, China	CIB 119043	OR189183	This study
6	*A.yunkaiensis*	Dawuling Forestry Station, Guangdong, China	SYS r001443	MN380329	Wang et al. (2019)
7	*A.yunkaiensis*	Dawuling Forestry Station, Guangdong, China	SYS r001502	MN380330	Wang et al. (2019)
8	*A.yunkaiensis*	Dawuling Forestry Station, Guangdong, China	SYS r001503	MN380331	Wang et al. (2019)
9	*A.yunkaiensis*	Dawuling Forestry Station, Guangdong, China	SYS r001902	MN380332	Wang et al. (2019)
10	*A.yunkaiensis*	Dawuling Forestry Station, Guangdong, China	SYS r001903	MN380333	Wang et al. (2019)
11	*A.yunkaiensis*	Maoershan Nature Reserve, Guangxi, China	YBU 14612	MT365525	Yu et al. (2020)
12	*A.yunkaiensis*	Xinning, Hunan, China	CIB 119041	OQ978852	Ma et al. (2023a) (in press)
13	*A.ater*	Huaping Nature Reserve, Guangxi, China	SYS r00852	MN380334	Wang et al. (2019)
14	*A.dabieshanensis*	Yaoluoping Nature Reserve, Anhui, China	AHU2018EE0710	MW316598	Zhang et al. (2023)
15	*A.damingensis*	Nanning, Guangxi, China	ANU20220009	OP644487	Yang et al. (2023)
16	*A.dehuaensis*	Dehua, Fujian, China	YBU 13013	MZ442642	Li et al. (2021)
17	*A.emilyae*	Dong Son-Ky Thuong Nature Reserve, Hoanh Bo, Vietnam	IEBR 4465	MK330857	Ziegler et al. (2019)
18	*A.formosanus*	Taiwan, China	RN2002	KU529452	Unpublished
19	*A.hunanensis*	Huaihua, Hunan, China	CIB 119039	OQ848425	Ma et al. (2023b)
20	*A.hunanensis*	Ningxiang, Hunan, China	CIB 119040	OQ848426	Ma et al. (2023b)
21	*A.huangjietangi*	Huangshan, Anhui, China	HSR18030	MT380191	Huang et al. (2021)
22	*A.juliani*	Ha Lang, Cao Bang, Vietnam	IEBR A.2018.8	MK330854	Ziegler et al. (2019)
23	*A.meiguensis*	Mianyang, Sichuan, China	GP835	MZ442641	Li et al. (2021)
24	*A.niger*	Taiwan, China	RN0667	KU529433	Unpublished
25	*A.ningshanensis*	Ningshan, Shaanxi, China	ANU 20220006	ON548422	Yang et al. (2022)
26	*A.panzhihuaensis*	Yanbian, Sichuan, China	KIZ 040189	MW664862	Hou et al. (2021)
27	*A.pingbianensis*	Honghe, Yunnan, China	YBU 18273	MT365521	Li et al. (2021)
28	*A.quangi*	Phu Yen, Son La, Vietnam	ZVNU.2022.08	OQ197471	Pham et al. (2023)
29	*A.rufescens*	Hongkong, China	SYS r001866	MN380339	Wang et al. (2019)
30	*A.spinalis*	Badagong Mountains, Hunan, China	SYS r001327	MN380340	Wang et al. (2019)
31	*A.timi*	Thuan Chau, Son La, Vietnam	IEBR A.2018.10	MK330856	Ziegler et al. (2019)
32	*A.tranganensis*	Ninh Binh, Vietnam	VNUF R.2018.21	MW023086	Luu et al. (2020)
33	*A.vanhoensis*	Van Ho, Son La, Vietnam	VNUF R.2019.13	ON677935	Ha et al. (2022)
34	*A.yangdatongi*	Wenshan Nature Reserve, Yunnan, China	KIZ 034327	MW664865	Hou et al. (2021)
35	*A.zugorum*	Bac Me, Ha Giang, Vietnam	IEBR 4698	MT502775	Miller et al. (2020)
36	*Fimbriosklossi*	Quang Ngai, Vietnam	IEBR 3275	KP410744	Teynié et al. (2015)
37	*Parafimbrioslao*	Louangphabang, Laos	MNHN 2013.1002	KP410746	Teynié et al. (2015)
38	*Xenodermusjavanicus*	Sumatera Barat, Sumatra, Indonesia	–	KP410747	Teynié et al. (2015)

*CO1* sequences (618 bp) were input in MEGA11 (Tamura et al. 2021) and aligned by MUSCLE (Edgar 2004). Then we calculated the uncorrected pairwise distances (*p*-distance) in MEGA11. IQ-TREE 1.6.12 was performed to conduct the Maximum Likelihood (ML) analysis (Nguyen et al. 2015) under the best-fit model TN+F+I+G4 computed by ModelFinder according to Bayesian Information Criterion (BIC) (Kalyaanamoorthy et al. 2017). Ultrafast Bootstrap Approximation (UFB) node support was assessed by using 5000 ultrafast bootstrap replicates and the UFB (%) ≥ 95 was considered significantly supported (Hoang et al. 2018). The single branch tests were conducted by SH-like approximate likelihood ratio test (SH-aLRT) by 1000 replicates and the nodal support (SH, %) ≥ 80 was also considered supported well (Stephane et al. 2010). The Bayesian Inference (BI) analysis was conducted via MrBayes (Ronquist et al. 2012) in PhyloSuite 1.2.3 (Zhang et al. 2020) by using a four chains run calculated for 10 million generations under the best model TN+F+I+G4, sampling every 1000 with the first 25% of samples discarded as burn-in and the nodal support Bayesian posterior probabilities (BI, %) ≥ 95 were considered significantly supported.

### ﻿Morphological characters

Morphological data were obtained from the five newly-collected specimens, examination of museum specimens (Appendix 1) and many key references (Boulenger 1888, 1893, 1896; Denburgh 1912; Bourret 1935, 1937; Hu and Zhao 1966; Hu et al. 1973; Koshikawa 1982; Zong and Ma 1983; Ota and Toyama 1989; Zhao et al. 1998; Zhao 2006; Wang et al. 2019; Ziegler et al. 2019; Li et al. 2020; Luu et al. 2020; Miller et al. 2020; Yu et al. 2020; Hou et al. 2021; Huang et al. 2021; Li et al. 2021; Chen et al. 2022; Ha et al. 2022; Yang et al. 2022; Li et al. 2023; Ma et al. 2023a, b; Pham et al. 2023; Xu et al. 2023; Yang et al. 2023; Zhang et al. 2023).

Morphological descriptions followed Zhao (2006) and Ma et al. (2023b): three measurement characters were measured to the nearest 0.1 mm using a Deli Stainless Ruler (No. 8460): snout-vent length (**SVL**), tail length (**TaL**) and total length (**TL**); other measurement characters were measured to the nearest 0.01 mm using a Deli Digital Vernier Caliper (DL91150): head length (**HL**), head width (**HW**), eye horizontal diameter (**ED**), loreal height (**LorH**), loreal length (**LorL**), length of the suture between internasals (**LSBI**), length of the suture between prefrontals (**LSBP**), length of supraocular (**SPOL**: horizontal distance between anterior and posterior tip of supraocular) and length of upper anterior temporal (**ATUL**: horizontal distance between anterior and posterior tip of upper anterior temporal). We also directly compared the length of the sutures between internasals and prefrontals (**LSBI** vs. **LSBP**). Scalation features and their abbreviations are as follows: loreals (**Loreal**), supralabials (**SPL**), infralabials (**IFL**), the number of infralabials touching the first pair of chin shields (**IFL-1^st^ Chin**), supraoculars (**SPO**), temporals (**TEM**), the number of anterior temporals touching the eye (**aTEM-Eye**), ventral scales (**VEN**), subcaudal (**SC**), entire or divided of the cloacal plate (**Anal**), dorsal scale rows (**DSR**) (counted at one-head-length behind the head, at midbody, at one-head-length before the cloacal plate). We also counted the number of maxillary teeth (**MT**) under the microscope. Bilateral scale counts were given as left/right.

## ﻿Results

### ﻿Molecular systematics

**Figure 2.**
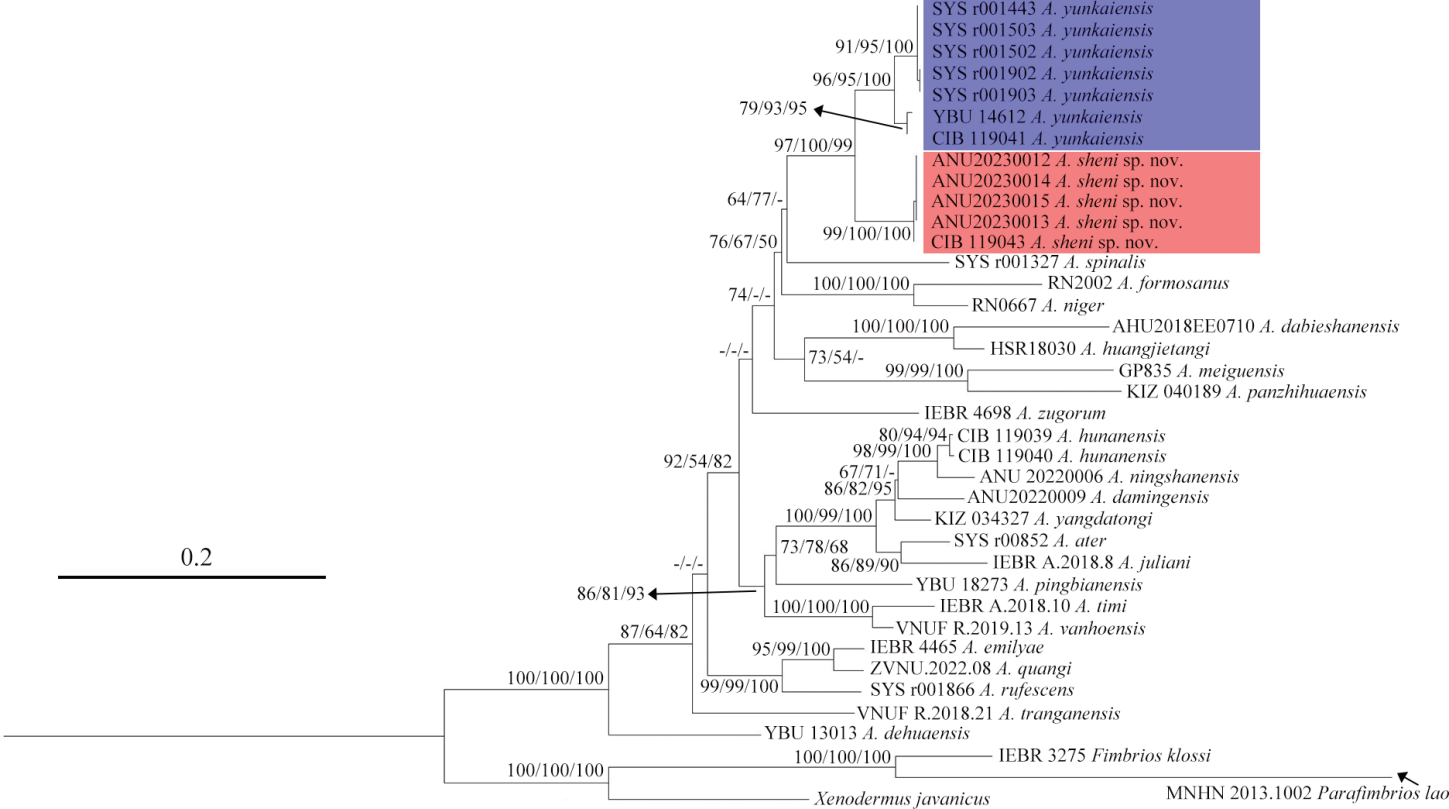
Phylogenetic tree of the genus *Achalinus* inferred from the *CO1* gene fragment (618 bp) using Maximum Likelihood. The support values of each node present on the tree: SH / UFB / BI (the ones lower than 50 are displayed as “-”). *A.yunkaiensis* is noted in blue and *A.sheni* sp. nov. is noted in red.

The unnamed *Achalinus* specimens form a sister lineage (SH 99/UFB 100/BI 100) to the species *A.yunkaiensis* Wang, Li & Wang, 2019 (SH 96/UFB 95/BI 100) with a significantly high nodal support (SH 97/ UFB 100/BI 99) (Fig. 2).

Amongst the *Achalinus* species studied in this work, the genetic distances inferred from the mitochondrial *CO1* gene fragment range from 3.2% (*A.hunanensis* Ma, Shi, Xiang, Shu & Jiang, 2023 vs. *A.ningshanensis* Yang, Huang, Jiang, Burbrink, Gong, Yu, Zhang, Huang & Huang, 2022) to 18.1% (*A.meiguensis* Hu & Zhao, 1966 and *A.dehuaensis* Li, Wu, Xu, Zhu, Ren, Guo & Dong, 2021), while the genetic distances between the lineage formed by the newly-collected *Achalinus* specimens and its congeners range from 5.8% (vs. *A.yunkaiensis*) to 15.8% (vs. *A.dabieshanensis* Zhang, Liu, Huang & Zhang, 2023), indicating that these newly-collected specimens have distinct genetic differentiation from the other *Achalinus* species (Table 2).

**Table 2.** Uncorrected *p*-distances (%) amongst *Achalinus* species, based on the mitochondrial *CO1* gene.

	1–5	6–12	13	14	15	16	17	18	19–20	21	22	23	24	25	26	27	28	29	30	31	32	33	34
1–5 *A.sheni* sp. nov.	0–0.2																						
6–12 *A.yunkaiensis*	5.8–6.4	0–2.8																					
13 *A.ater*	12.8–12.9	11.5–12.9																					
14 *A.dabieshanensis*	15.6–15.8	14.9–15.8	14.7																				
15 *A.damingensis*	13.6–13.8	12.3–12.6	7.9	15.8																			
16 *A.dehuaensis*	13.4–13.6	13.9–14.7	16.3	18.4	16.0																		
17 *A.emilyae*	13.1	12.4–13.3	11.5	17.7	12.8	15.2																	
18 *A.formosanus*	12.8–12.9	12.2–12.6	13.9	19.0	14.9	15.7	13.8																
19–20 *A.hunanensis*	12.0–12.5	12.5–13.1	7.1–7.3	16.9–17.1	6.1–6.3	15.1–15.3	13.0–13.3	13.8–14.0	0.5														
21 *A.huangjietangi*	13.3–13.5	12.1–12.5	15.0	8.9	16.2	16.4	14.1	15.3	16.8–16.9														
22 *A.juliani*	13.6–13.8	11.4–12.3	7.0	15.8	8.4	14.7	12.3	12.5	8.7–8.8	14.4													
23 *A.meiguensis*	13.9–14.1	12.2–13.1	15.4	17.7	16.8	18.1	15.4	15.6	16.4	15.2	16.8												
24 *A.niger*	12.3–12.5	12.2–12.6	13.6	15.8	14.1	15.7	12.0	8.9	13.3	13.9	12.3	13.9											
25 *A.ningshanensis*	14.1–14.2	15.2–16.0	7.5	17.2	7.7	16.2	14.1	14.8	3.2–3.3	17.0	9.7	17.0	14.6										
26 *A.panzhihuaensis*	14.6	10.5–12.3	16.2	16.6	15.5	15.3	16.6	16.0	16.2	15.2	15.5	11.6	14.4	17.4									
27 *A.pingbianensis*	11.5–11.6	12.8–13.9	11.8	15.3	11.3	14.9	13.0	14.6	11.2	13.0	12.1	16.8	11.8	11.7	14.9								
28 *A.quangi*	13.9	15.5–15.8	11.5	18.1	12.9	15.0	3.6	13.8	13.1–13.2	14.6	12.6	15.2	11.7	13.3	16.9	13.9							
29 *A.rufescens*	12.9	11.6–12.1	12.5	16.9	13.6	13.9	8.1	13.9	12.1–12.2	13.9	12.3	17.3	12.5	12.2	16.0	13.0	7.9						
30 *A.spinalis*	11.2–11.3	12.2–13.6	15.2	16.6	15.0	14.1	13.9	13.9	14.0–14.3	13.1	14.1	16.0	13.4	15.7	15.8	13.3	13.9	12.9					
31 *A.timi*	13.6–13.8	11.7–13.3	13.1	16.4	13.3	16.0	12.8	13.6	12.1–12.4	14.6	13.9	15.8	11.8	13.3	15.5	12.3	13.3	13.6	14.1				
32 *A.tranganensis*	13.3	11.7–12.1	12.6	15.3	13.9	13.8	11.5	16.8	13.8–14.2	13.1	13.4	16.4	14.2	15.3	16.4	13.3	12.1	11.5	14.7	13.6			
33 *A.vanhoensis*	13.4–13.6	13.0–14.1	12.7	15.5	12.3	15.7	12.2	13.9	11.3–11.7	14.2	13.4	15.6	12.3	11.9	15.5	10.8	12.3	13.7	12.7	4.7	13.0		
34 *A.yangdatongi*	13.7–13.8	12.5–13.6	6.2	16.6	5.6	14.0	12.8	14.4	5.1	14.6	7.3	17.1	13.7	5.9	15.5	11.3	12.6	11.5	14.2	13.1	12.8	11.3	
35 *A.zugorum*	10.4–10.5	12.3–13.4	12.9	15.3	12.8	14.1	12.1	13.3	11.7–12.0	14.1	13.1	15.0	12.9	12.8	15.3	11.0	12.5	13.3	13.3	13.4	11.8	11.8	12.2

Based on the molecular results above, these specimens are supported to be an unnamed taxon.

### ﻿Morphological characters

**Figure 3.**
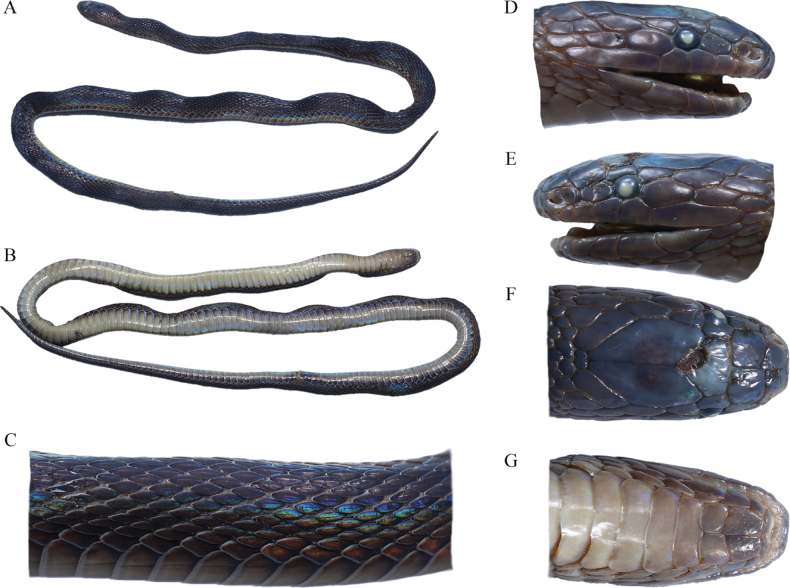
Holotype (ANU20230014, adult male) of *Achalinussheni* sp. nov. **A** dorsolateral view **B** ventral view **C** right side of middle body view **D** light side of head view **E** right side of head view **F** dorsal head view **G** ventral head view. Photos by Yu-Hao Xu.

**Figure 4.**
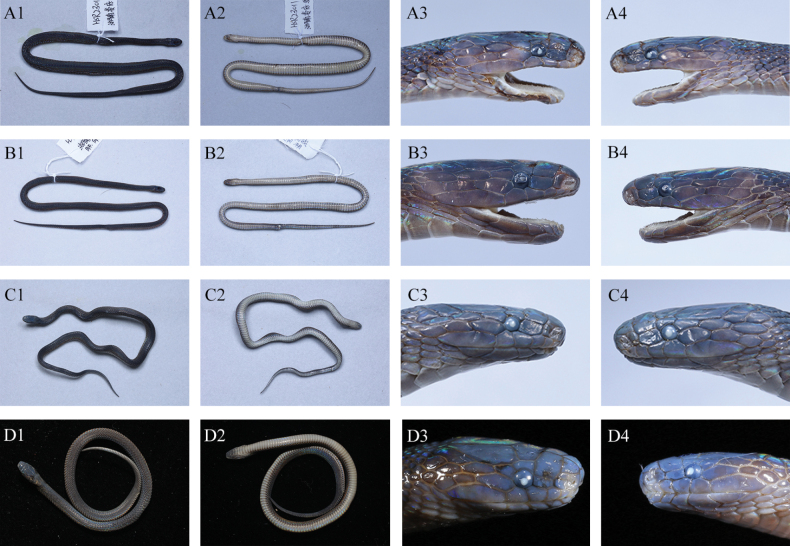
Paratypes of *A.sheni* sp. nov. **A** ANU20230012 (adult male) **B** ANU20230013 (adult male) **C** ANU20230015 (subadult male) **D**CIB 119043 (juvenile male). **A–C** photos by Yu-Hao Xu, **D1** and **D2** photos by Ke-Ji Guo, **D3** and **D4** photos by Sheng-Chao Shi.

**Figure 5.**
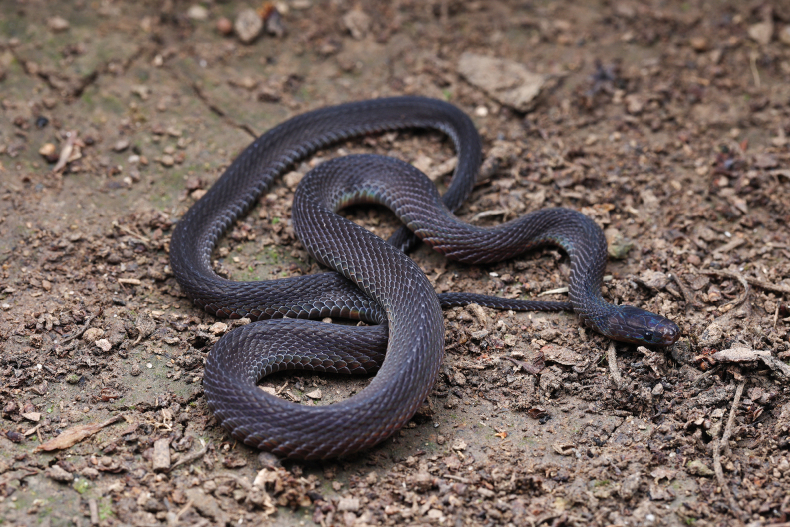
Paratype (ANU20230013, adult male) of *A.sheni* sp. nov in life. Photos by Yu-Hao Xu.

**Figure 6.**
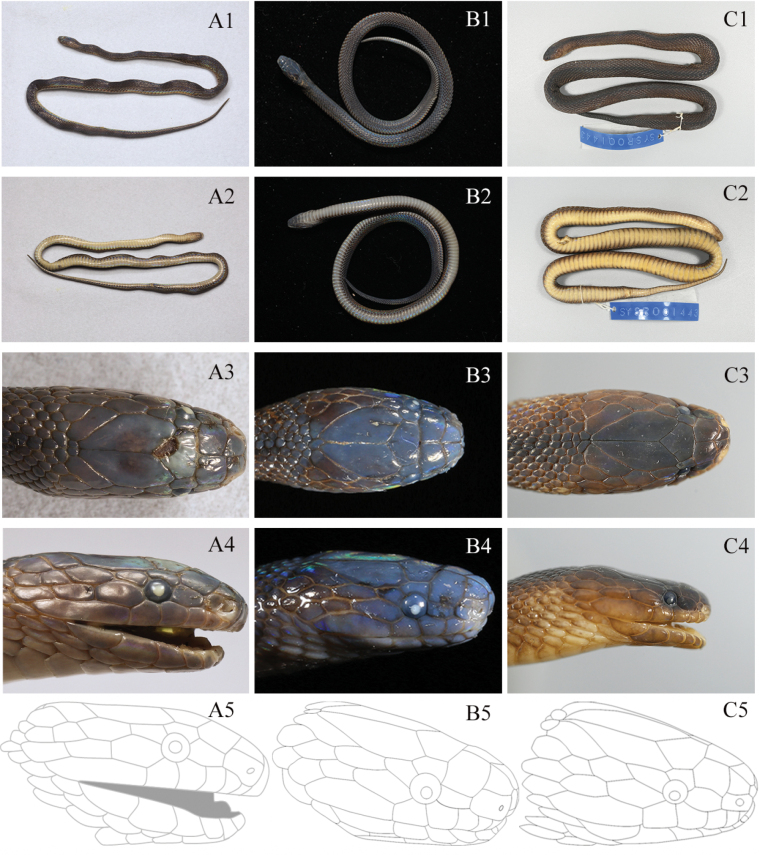
Scalation and colouration comparisons between *Achalinussheni* sp. nov. and *A.yunkaiensis***A***A.sheni* sp. nov. (ANU20230014, adult male), **A1–A4** photos by Yu-Hao Xu **B***A.sheni* sp. nov. (CIB 119043, juvenile male), **B1–B2** photos by Ke-Ji Guo and **B3–B4** photos by Sheng-Chao Shi **C***A.yunkaiensis* (SYS r001443, adult male): **C1–C4** photos by Shuo Qi. Line illustration (**A5, B5** and **C5**) by Jie-Fang Chen.

The five newly-collected *Achalinus* specimens from Hunan Province can be easily distinguished from all other known congeners (Table 3, 4, Figs 3–5). By internasal separated from prefrontal, they differ from *A.meiguensis* (vs. internasal fused to prefrontal) and *A.panzhihuaensis* Hou, Wang, Guo, Chen, Yuan & Che, 2021 (vs. internasal fused to prefrontal). By having LSBI vs. LSBP = 1, they differ from *A.ater* (vs. > 1), *A.dabieshanensis* (vs. > 1), *A.damingensis* Xu, Yang, Wu, Gong, Huang & Huang, 2023 (vs. > 1), *A.dehuaensis* (vs. > 1), *A.emilyae* Ziegler, Nguyen, Pham, Nguyen, Pham, van Schingen, Nguyen & Le, 2019 (vs. > 1), *A.huangjietangi* (vs. < 1), *A.hunanensis* (vs. > 1), *A.jinggangensis* (Zong & Ma, 1983) (vs. > 1), *A.juliani* Ziegler, Nguyen, Pham, Nguyen, Pham, van Schingen, Nguyen & Le, 2019 (vs. > 1), *A.niger* (vs. < 1), *A.quangi* Pham, Pham, Le, Ngo, Ong, Ziegler & Nguyen, 2023 (vs. > 1), *A.rufescens* (vs. > 1), *A.spinalis* (vs. < 1), *A.timi* Ziegler, Nguyen, Pham, Nguyen, Pham, Van Schingen, Nguyen & Le, 2019 (vs. > 1), *A.tranganensis* Luu, Ziegler, Ha, Lo, Hoang, Ngo, Le, Tran & Nguyen, 2020 (vs. > 1), *A.yangdatongi* Hou, Wang, Guo, Chen, Yuan & Che, 2021 (vs. > 1), *A.vanhoensis* Ha, Ziegler, Sy, Le, Nguyen & Luu, 2022 (vs. > 1) and *A.zugorum* Miller, Davis, Luong, Do, Pham, Ziegler, Lee, De Queiroz, Reynolds & Nguyen, 2020 (vs. > 1). By loreal separated from prefrontal, they are different from *A.formosanuschigirai* Ota & Toyama, 1989 (vs. loreal fused to prefrontal), *A.f.formosanus* Boulenger, 1908 (vs. loreal fused to prefrontal) and *A.pingbianensis* Li, Yu, Wu, Liao, Tang, Liu & Guo, 2020 (vs. loreal fused to prefrontal). By TaL/TL 0.183 ~ 0.224, they can differ from *A.hainanus* (vs. 0.258 ~ 0.266), *A.ningshanensis* (vs. 0.121 ~ 0.161) and *A.werneri* (vs. 0.250 ~ 0.300). They also can be easily distinguished from their sister taxon *A.yunkaiensis* by the following morphological characters: (1) relative length of supraocular and upper anterior temporal (supraocular equal to or longer than anterior temporal, SPOL/ATUL 0.99 ~ 1.20 vs. supraocular shorter than anterior temporal, SPOL/ATUL 0.55 ~ 0.83); (2) more ventral scales + subcaudals counts in males (220–225 vs. 200–212); (3) more ventral scales in males (161–170 vs. 150–162); (4) more subcaudals in males (55–61 vs. 49–56); (5) less infralabials (5 (rarely 6) vs. 6); (6) more maxillary teeth in males (24 vs. 20–21); and (7) different uniform dorsal colouration pattern (dark brown vs. brown) (Table 5, Fig. 6).

**Table 3.** Main morphological characters of *Achalinussheni* sp. nov.

Voucher Number	ANU20230014	ANU20230012	ANU20230013	ANU20230015	CIB 119043
	Holotype	Paratype	Paratype	Paratype	Paratype
**Sex**	**Adult male**	**Adult male**	**Adult male**	**Subadult male**	**Juvenile male**
**SVL**	292.2	278.8	253.8	208.7	121.8
**TaL**	79.1	80.3	67.9	56.7	27.2
**TL**	371.3	359.1	321.7	265.4	149.0
**TaL/TL**	0.213	0.224	0.211	0.214	0.183
**HW**	5.13	5.39	4.34	4.24	3.28
**HL**	10.74	11.28	10.62	9.26	6.96
**ED**	1.25	1.26	1.17	0.94	0.88
**MT**	–	–	–	–	24
**SPL**	6/6	6/6	6/6	6/6	6/6
**SPL-Eye**	4^th^–5^th^	4^th^–5^th^	4^th^–5^th^	4^th^–5^th^	4^th^–5^th^
**IFL**	5/5	5/5	5/5	6/5	5/5
**Chin**	2	2	2	2	2
**IFL-1^st^ Chin**	1^st^–3^rd^	1^st^–3^rd^	1^st^–3^rd^	1^st^–3^rd^	1^st^–3^rd^
**Loreal**	1	1	1	1	1
**LorH**	0.85	0.93	0.83	0.83	0.69
**LorL**	1.49	1.71	1.54	1.45	1.29
**LorH/LorL**	0.57	0.54	0.54	0.57	0.53
**LSBI vs. LSBP**	=	=	=	=	=
**SPO**	1	1	1	1	1
**SPOL**	1.59	1.52	1.39	1.21	1.64
**TMP**	2+2+3	2+2+3	2+2+3	2+2+3	2+2+3
**ATMP-Eye**	2	2	2	2	2
**ATUL**	1.42	1.48	1.41	1.20	1.42
**SPOL/ATUL**	1.12	1.03	0.99	1.01	1.16
**DSR**	23-23-23	23-23-23	23-23-23	23-23-23	23-23-23
**V**	161	161	166	162	170
**SC**	60	61	57	58	55
**Anal**	1	1	1	1	1

**Table 4.** Morphological characters of *Achalinus* obtained from specimens examined in this study and literature. Int. fus.: internasal fused to prefrontal; Pre fus.: prefrontal fused to loreal; PtO: postoculars.

Species	TaL/TL	MT	Int fus.	Pre fus.	LorH/LorL	LSBI vs. LSBP	DSR	PtO	SPL	SPL-Eye	IFL	IFL-1^st^ Chin	TEM	aTEM-Eye	VEN	SC	Reference
*A.sheni* sp. nov.	0.183~0.224	24	0	0	0.53~0.57	=1	23-23-23	0	6	4–5	5–6	1–3	2+2+3	2	161–170	55–61	This study
*A.ater*	0.190~0.220	–	0	0	0.40	>1	(21–23)-(21–25)-(21–25)	0	6	4–5	5–6	1–3	2+2+3	2	160–170	47–70	Bourret (1935, 1937); Hu et al. (1973); Zhao et al. (1998); Zhao (2006)
*A.dabieshanensis*	0.168~0.223	–	0	0	0.73~0.83	>1	23-23-23	0	6	4–5	5	1–3	2+2+3(4)	2	141–155	45–55	Zhang et al. (2023)
*A.damingensis*	0.246	–	0	0	0.65	>1	23-23-23	0	6	4–5	6	1–3	2+2+3	2	162	74	Yang et al. (2023)
*A.dehuaensis*	0.206~0.286	31–33	0	0	–	>1	23-23-23	0	6	4–5	5	1–3	2+2(3)+3(4)	1–2	142–154	63–81	Li et al. (2021)
*A.emilyae*	0.183~0.203	27–28	0	0	–	>1	23-23-23	0	6	4–5	5	1–3	2+2+3	1	157–161	56–63	Ziegler et al. (2019); Hou et al. (2021)
*A.formosanuschigirai*	0.317	14	0	1	–	=1	(25–27)-(25–27)-25	0	6	4–5	5–6	–	2+2	2	161–167	96–97	Ota and Toyama (1989)
*A.f.formosanus*	0.159	17	0	1(usually)	–	=1	29-27-25	0	6	4–5	6–7	–	2+2	1	158–184	61–83	Ota and Toyama (1989)
*A.hainanus*	0.258~0.266	–	0	0	–	=1	23-23-23	0	6	4–5	5	1–3	1+2+3(4)	1	165–168	67–69	Koshikawa (1982); Zhao et al. (1998)
*A.huangjietangi*	0.152~0.232	–	0	0	0.70~0.74	<1	23-23-23	0	6	4–5	5–6	1–3(4)	2+2+3(4)	2	157–170	40–67	Huang et al. (2021); Chen et al. (2022)
*A.hunanensis*	0.221~0.225	23	0	0	0.62~0.70	>1	23-23-23	0	6	4–5	5–6	1–3(4)	2+2+4	2	163–165	69–72	Ma et al. (2023b)
*A.jinggangensis*	0.174~0.217	–	0	1	–	>1	23-23-23	0	6	4–5	6	1–4	2(1)+2+3(4)	2	156–164	51–64	Zong and Ma (1983); Zhao et al. (1998)
*A.juliani*	0.224~0.268	28	0	0	–	>1	25-23-23	0	6(7)	4–5(5–6)	6	1–3(4)	2+2+4	2	163–179	77–91	Ziegler et al. (2019)
*A.meiguensis*	0.142~0.238	17	1	0	–	–	(21–23)-(19–21)-(19–21)	1	6	4–5	6	1–3	2(3)+2(3)	1	146–173	39–60	Hu and Zhao (1966)
*A.niger*	0.151~0.179	–	0	0	0.67	<1	25-25-23	0	6	4–5	6	1–3(4)	2+2(3)	2	169–185	52–72	Ota and Toyama (1989); Zhao et al. (1998); Zhao (2006)
*A.ningshanensis*	0.121~0.161	–	0	0	0.45~0.58	=1	23-23-23(21)	0	6	4–5	5	1–2(3)	2+2(3)+3(4)	1–2	159–174	41–46	Yang et al. (2022)
*A.panzhihuaensis*	0.246	28	1	0	0.67	–	23-23-19	1	6	4–5	6	1–3	2+2+3	1	160	73	Hou et al. (2021)
*A.pingbianensis*	0.243	–	0	1	–	=1	23-23-23	0	7	5–6	6	1–3	2+2+3	1	164	56	Li et al. (2020)
*A.quangi*	0.219~0.304	27–29	0	0	–	>1	(23–25)-23-(21–23)	0	6	4–5	5	1–3	2+2+4	1–2	139–154	69–84	Pham et al. (2023)
*A.rufescens*	0.191~0.276	23	0	0	0.80~1.00	>1	23-(23–25)-23	0	6	4–5	5	1–3	2(1)+2+3(4)	1–2	132–156	58–82	Boulenger (1888, 1893, 1896); Wang et al. (2019)
*A.spinalis*	0.150~0.250	16–20	0	0	–	<1	(23–25)-(23–25)-(23–25)	0	6	4–5	5–6	1–3	2+2(3)	1–2	138–175	48–67	Zhao et al. (1998); Hou et al. (2021); Ha et al. (2022)
*A.timi*	0.213	27	0	1	–	>1	25-25-23	0	6	4–5	6	1–3	2+2+3	1	170	72	Ziegler et al. (2019)
*A.tranganensis*	0.254(+)	29	0	0	–	>1	25-23-23	0	6	4–5	6	1–3	2+2+3	2	171	73(+)	Luu et al. (2020)
*A.werneri*	0.250~0.300	–	0	0	–	=1	?-(21–23)-?	0	6	4–5	6	–	2+3(4)	–	157–191	67–98	Denburgh (1912); Ota and Toyama (1989)
*A.yangdatongi*	0.180~0.262	24–26	0	0	0.57	>1	23-23-23	0	6	4–5	5–6	1–3	2+2/3+2/3	2	155–171	59–76	Hou et al. (2021); Xu et al. (2023)
*A.yunkaiensis*	0.156~0.204	20–24	0	0	0.49~0.64	=1	23-23-23	0	6	4–5	6	1–3(4)	2+2+3(4)	2	144–162	49–56	Wang et al. (2019); Yu et al. (2020); Li et al. (2023); Ma et al. (2023a)
*A.vanhoensis*	0.264	32	0	1	–	>1	25-23-23	0	6/7	4–5/5–6	6	1–4	2+2+3	2	176	84	Ha et al. (2022)
*A.zugorum*	0.229	28	0	1	–	>1	25-23-23	0	6	4–5	7	1–3	2+2+3	2	173	70	Miller et al. (2020)

**Table 5.** Comparisons of main morphological characters of *Achalinussheni* sp. nov. and *A.yunkaiensis*.

Species	*A.sheni* sp. nov.	*A.yunkaiensis*	
N	5	4	3
Sex	♂	♂	♀
SVL	121.8–292.2	188.7–358.5	204–386.3
TaL	27.2–80.3	43.3–63.3	52–72.8
TL	149.0–371.3	232–417.6	256–448.1(+)
TaL/TL	0.183 ~ 0.224	0.185 ~ 0.200	0.156 ~ 0.204
MT	**24**	**20–21**	22–24
SPL	6	6	6
SPL-Eye	4^th^–5^th^	4^th^–5^th^	4^th^–5^th^
IFL	**5 (rarely 6)**	**6**	**6**
IFL-1^st^ Chin	1^st^–3^rd^	1^st^–3^rd^	1^st^–3^rd^/4^th^
Loreal	1	1	1
LorH	0.69–0.93	0.8–1.3	0.74–1.2
LorL	1.29–1.71	1.3–2.2	1.51–2.2
LorH/LorL	0.53 ~ 0.57	0.56 ~ 0.64	0.49 ~ 0.55
LSBI vs. LSBP	=	=	=
SPO	1	1	1
SPOL	1.21–1.59	0.97–1.62	1.26–1.60
TMP	2+2+3	2+2+3/4	2+2+3/4
ATMP-Eye	2	2	2
ATUL	1.20–1.48	1.18–2.18	1.93–2.90
SPOL/ATUL	**0.99** ~ **1.16**	**0.66** ~ **0.83**	**0.55** ~ **0.65**
DSR	23-23-23	23-23-23	23-23-23
V	**161**–**170**	**151–162**	**144–156**
SC	**55**–**61**	**49**–**56**	**51–55**
V + SC	**220–225**	**200–212**	**195–205**
Anal	1	1	1
References	This study	Wang et al. (2019)	Wang et al. (2019); Yu et al. (2020); Ma et al. (2023a)

Therefore, combining the results of molecular systematics and morphological characters mentioned above, these five specimens, newly collected from Hunan Province, represent a new species and we describe it herein.

### ﻿Taxonomic account

#### ﻿ Achalinus sheni sp. nov.

Taxon classificationAnimaliaSquamataXenodermidae

903D9271-AA1B-5936-B194-115111BEC5E4

https://zoobank.org/7FBF50AF-C1D8-46C6-8B66-A5805598AFF8

Figs 3 , 4 , 5 , 6

##### Chresonymy.

*Achalinusspinalis*: Li et al. (2010).

##### Type material.

***Holotype*.** ANU20230014 (field number HSR23019, Fig. 3), an adult male, collected on 21 March 2023 (27°55′11″N, 111°55′3″E; 408 m a. s. l.), Qixingjie Town, Lianyuan City, Hunan Province, China by the team of Song Huang.

***Paratypes*.** Three males, ANU20230012 (field number HSR23011, Fig. 4A), ANU20230013 (field number HSR23012, Figs 4B, 5), ANU20230015 (subadult male, field number HSR23020, Fig. 4C), with the same collecting information as the holotype; CIB 119043, a juvenile male, collected on 20 October 2015 by Bing Zhou and Shanshan Tang from Shumuyuan, Nanyue District, Hunan Province, China (27°15′59″N, 112°43′15″E; 358 m a.s.l., Fig. 4D).

##### Etymology.

The species name “*sheni*” is named for the memories of the Chinese herpetologist, Prof. You-Hui Shen (沈猷慧), who worked in Hunan Normal University and made great contributions to the herpetological research of China, particularly in Hunan Province where the new species is found. We suggest “Shen’s Odd-scale Snake” or “Shen’s Burrowing Snake” as its English name and “沈氏脊蛇” (Shěn Shì Jǐ Shé) as its Chinese name.

##### Diagnosis.

(1) dorsal scales strongly keeled, 23 rows throughout the body, the outmost row smooth and significantly enlarged ; (2) tail relatively short, TaL/TL 0.183 ~ 0.224; (3) the suture between internasals subequal to the suture between prefrontals; (4) loreal one, subrectangular, LorH/LorL 0.53 ~ 0.57; (5) ventrals 161–170, anal entire, subcaudals 55–61, not paired; (6) the length of supraocular equal to or longer than the length of upper anterior temporal; (7) vertebral line inconspicuous and subcaudal streak absent.

##### Description of holotype.

An adult male with a total length of 371.3 mm (SVL 292.2 mm and TaL 79.1 mm); tail relatively short, Tal/TL 0.213; body slender, cylindrical; head length (HL) 10.74 mm, head width 5.13 mm, HL/HW 2.09, slightly distinct from neck; eye small, ED 1.25 mm, with an oval pupil; maxillary teeth 21. Rostral small, triangular, only the upper tip visible from above. Length of the suture between the internasals (LSBI 1.38 mm) subequal to the length of the suture between the prefrontals (LSBP 1.36 mm). Nostril in the anterior part of the nasal. Loreal one, subrectangular, loreal height (LorH) 0.85 mm, loreal length (LorL) 1.49 mm, LorH/LorL 0.57. Frontal one, pentagonal, pointed backwards, much shorter than the parietals. Parietals paired. No preoculars and postoculars. Supraocular one, length of supraocular (SPOL 1.59 mm) longer than the length of upper anterior temporal (ATUL 1.42 mm, SPOL/ATUL 1.12). Temporals 2+2+3, the anterior two contact the eye, the lower anterior temporal much larger, the upper medium temporal much larger, the upper posterior temporal much larger and separated from the other side one by one scale. Supralabials 6, 4^th^–5^th^ contact the eye, the last one much elongated. One mental. Two chin shields, the anterior pairs longer than the posterior pairs. Infralabials 5, the first one contact with each other after the mental and before the 1^st^ chin shields, 1^st^–3^rd^ touch the 1^st^ chin shields.

Dorsal scales strongly keeled, 23 rows throughout the body, the outmost row smooth and significantly enlarged. Ventrals 161; anal entire; subcaudals 60, not paired.

##### Colouration of holotype in life.

Scales tinged weakly iridescent and metallic lustre. Dorsum dark brown and the five innermost dorsal scale rows a little darker, forming an inconspicuous longitudinal vertebral line. Chin shields are tan. Ventrals generally light brown, darker on both sides, free margins of ventral scales greyish-white. Ventral side of tail brownness.

##### Colouration of holotype in preservation.

The dorsal surface of the body uniformly brownish-black, slightly tinged with iridescence and the longitudinal vertebral line a little darker. Chin shields light brown. Ventrals generally creamy-brown, darker on both sides, free margins of ventral scales greyish-white. Ventral side of tail light brown.

##### Variation.

Measurements, body proportions and scale counts are listed in Table 3. All paratypes are very similar to the holotype, except in the following: (1) paratype ANU20230004 has six infralabials on the left side; (2) relatively shorter supraoculars (SPOL/ATUL): ANU20230012: 1.03, ANU 20230013: 0.99, ANU20230004: 1.01; (3) more ventrals: ANU 20230013: 166, CIB 119043: 170; (4) less subcaudals: ANU 20230013: 57, ANU20230015: 58, CIB 119043: 55; (5) dorsum dark brown, venter greyish-white, both sides of ventral scales are taupe and ventral view of tail light grey in both subadult male ANU20230015 and juvenile male CIB 119043.

##### Distribution and habits.

*Achalinussheni* sp. nov. is currently only known from Hunan Province, China: Lianyuan City and Nanyue District (350–410 m a.s.l.). The native vegetation in the type locality is subtropical evergreen broad-leaved forests. Areas near the locality where the specimen CIB 119043 was collected is largely covered with artificial coniferous forest dominated by *Cryptomeria* spp. This new species’ population status requires further investigation. The conservation status for the new species is recommended to rate as data deficient (DD).

## ﻿Discussion

The description of *A.sheni* sp. nov. brings the total number of *Achalinus* known species to 27, with 20 species distributed in China, amongst which 16 species are endemic to China. Amongst these, four *Achalinus* species have been reported in the Hunan Province, *A.hunanensis* (the north mountain area in western Hunan Province), *A.spinalis* (the mountain area in north-western Hunan Province, the west mountainous and hilly areas in southern Hunan Province and the north mountainous and hilly areas in eastern Hunan Province), *A.jinggangensis* (the east mountainous and hilly areas in southern Hunan Province) and *A.yunkaiensis* (the southern mountain area in western Hunan Province) (Gao et al. 2022; Ma et al. 2023a) and the description of *A.sheni* sp. nov. (the hilly area in central Hunan Province) raises this number to five.

*Achalinus* is a group of poorly-known snakes as many species only have a single voucher specimen: *A.damingensis*, *A.panzhihuaensis*, *A.pingbianensis*, *A.timi*, *A.tranganensis*, *A.vanhoensis* and *A.zugorum* (Ziegler et al. 2019; Li et al. 2020; Luu et al. 2020; Miller et al. 2020; Hou et al. 2021; Ha et al. 2022; Yang et al. 2023) and several species (e.g. *A.hainanus* and *A.werneri*) do not have any sequence data accessioned. Consequently, this lack of adequate taxonomic sampling and genome-scale data results in our current situation where the population status, distribution pattern and evolution history of taxa within this genus are unclear (Miller et al. 2020). Therefore, it is paramount to conduct further survey work in these regions to learn more about these snakes.

### ﻿Key to species of the genus *Achalinus* Peters, 1869

**Table:** 

1	Internasal absent	**2**
–	Internasal present	**3**
2	Middle dorsal scale rows 23, subcaudal 39–62	***A.meiguensis***
–	Middle dorsal scale rows 19–21, subcaudal 73	***A.panzhihuaensis***
3	Loreal absent or usually absent	**4**
–	Loreal present	**9**
4	Middle dorsal scale rows 23	**5**
–	Middle dorsal scale rows ≥ 25	**7**
5	Dorsal scale rows 25-23-23	***A.vanhoensis***
–	Dorsal scale rows 23-23-23	**6**
6	Supralabials 6, internasal suture longer than prefrontal suture	***A.jinggangensis***
–	Supralabials 7, internasal suture subequal to prefrontal suture	***A.pingbianensis***
7	Internasal suture shorter than prefrontal suture	***A.formosanusformosanus***
–	Internasal suture longer than prefrontal suture	**8**
8	TaL/TL 0.317, ventrals 161–167, subcaudals 96–97	***A.formosanuschigirai***
–	TaL/TL 0.213, ventrals 170, subcaudals 72	***A.timi***
9	Anterior dorsal scale rows 25	**10**
–	Anterior dorsal scale rows usually 23	**13**
10	Dorsal scale rows 25-25-23	***A.niger***
–	Dorsal scale rows 25-23-23	**11**
11	Infralabials 7	***A.zugorum***
–	Infralabials 6	**12**
12	Prefrontals 2, ventrals 179 in female	***A.juliani***
–	Prefrontals 4, ventrals 171 in female	***A.tranganensis***
13	Internasal suture shorter than prefrontal suture	**14**
–	Internasal suture subequal to or longer than prefrontal suture	**15**
14	A dark streak in the middle of caudal ventral present	***A.huangjietangi***
–	A dark streak in the middle of caudal ventral absent	***A.spinalis***
15	Internasal suture subequal to prefrontal suture	**16**
–	Internasal suture longer than prefrontal suture	**20**
16	Anterior temporal 1	***A.hainanus***
–	Anterior temporal 2	**17**
17	TaL/TL 0.250 ~ 0.300, subcaudals 67–98	***A.werneri***
–	TaL/TL less than 0.225, subcaudals less than 61	**18**
18	The outmost dorsal scale rows keeled	***A.ningshanensis***
–	The outmost dorsal scale rows smooth	**19**
19	Ventrals 150–162, subcaudals 49–56, length of supraocular shorter than the length of upper anterior temporal	***A.yunkaiensis***
–	Ventrals 161–170, subcaudals 55–61, length of supraocular subequal to or longer than the length of upper anterior temporal	***A.sheni* sp. nov.**
20	Loreal elongate, length twice as height	***A.ater***
–	Loreal subquadrate, length longer than height, but the ratio less than two	**21**
21	Ventrals less than 156	**22**
–	Ventrals more than 155	**24**
22	Subcaudals less than 55	***A.dabieshanensis***
–	Subcaudals more than 58	**23**
23	Maxillary teeth 31–33	***A.dehuaensis***
–	Maxillary teeth 27–29	***A.quangi***
–	Maxillary teeth 23	***A.rufescens***
24	1 anterior temporal touching the eye	***A.emilyae***
–	2 anterior temporals touching the eye	**25**
25	TaL/TL 0.261 ~ 0.262 in males, ventrals 155 in males, subcaudals 76 in males	***A.yangdatongi***
–	TaL/TL 0.246 in male, ventrals 162 in male, subcaudals 74 in male	***A.damingensis***
–	TaL/TL 0.221 ~ 0.225 in males, ventrals 163–165 in males, subcaudals 69–72 in males	***A.hunanensis***

## Supplementary Material

XML Treatment for Achalinus sheni

## ﻿Additional information

### Author ORCIDs

Shun Ma https://orcid.org/0009-0003-8611-4550

Yu-Hao Xu https://orcid.org/0000-0001-6094-6680

Shuo Qi https://orcid.org/0000-0002-2924-6093

Shan-Shan Tang https://orcid.org/0009-0007-6582-3859

Song Huang https://orcid.org/0000-0001-6786-8523

Jian-Ping Jiang https://orcid.org/0000-0002-1051-7797

### Data availability

All of the data that support the findings of this study are available in the main text.

## ﻿Appendix 1. Specimens examined

Examined *Achalinus* specimens

*A.yunkaiensis* (n = 6): China

Xinyi City in Guangdong Province: SYS r001443, SYS r001502, SYS r001503,

SYS r001902, SYS r001903;

Xinning County in Hunan Province: CIB 119041.

## References

[B1] Boulenger GA (1888) Description of two new snakes from Hongkong, and note on the dentition of *Hydrophisviperina*. Annals & Magazine of Natural History, Series 6 7(2): 43–44. 10.1080/00222938809460874

[B2] Boulenger GA (1893) Catalogue of the Snakes in the British Museum (Nature History). I. London (Taylor & Francis), 448 pp.

[B3] Boulenger GA (1896) Catalogue of the Snakes in the British Museum (Natural History). III. London, 612 pp.

[B4] Bourret R (1935) Notes herpétologiques sur l‘Indochine française. VIII. Sur les *Achalinus* d’Indochine. Bulletin général de l’Instruction Publique, Hanoi, 14e Année (1934–1935) (5 janvier 1935): 101–104. [Separate: 1–4]

[B5] Bourret R (1937) Notes herpétologiques sur l’Indochine française. XV. Lézards et serpents reçus au Laboratoire des Sciences Naturelles de l’Université au cours de l’année 1937. Descriptions de deux espèces et de deux variétés nouvelles. Bulletin général de l’Instruction Publique, Hanoi, 17e Année (1937–1938), (4 Décembre 1937), Annexe, 57–80.

[B6] Chen CW Zhang CW Ding GH Zhang BW Wang YP (2022) A new snake record of *Achalinushuangjietangi* in Zhejiang Province, China. Yesheng Dongwu 43(4): 1149–1150. [In Chinese with English abstract]

[B7] Denburgh JV (1912) Concerning certain species of reptiles and amphibians from China, Japan, the Loo Choo Islands, and Formosa. Proceedings of the California Academy of Sciences 3(10): 187–258.

[B8] Edgar RC (2004) MUSCLE: Multiple sequence alignment with high accuracy and high throughput. Nucleic Acids Research 32(5): 1792–1797. 10.1093/nar/gkh340 15034147 PMC390337

[B9] Gao ZW Qian TY Jiang JP Hou DJ Deng XJ Yang DD (2022) Species diversity and distribution of amphibians and reptiles in Hunan Province, China. Shengwu Duoyangxing 30(2): 21290. 10.17520/biods.2021290

[B10] Ha NV Ziegler T Sy TD Le MD Nguyen TQ Luu VQ (2022) A new species of the genus *Achalinus* (Squamata: Xenodermidae) from Son La Province, Vietnam. Zootaxa 5168(3): 375–387. 10.11646/zootaxa.5168.3.8 36101279

[B11] Hoang DT Chernomor O Haeseler AV Minh BQ Vinh LS (2018) UFBoot2: Improving the ultrafast bootstrap approximation. Molecular Biology and Evolution 35(2): 518–522. 10.1093/molbev/msx281 29077904 PMC5850222

[B12] Hou SB Wang K Guo P Chen JM Yuan ZY Che J (2021) Two new species and a new country record of the genus *Achalinus* (Reptilia: Squamata: Xenodermidae) from China. Zootaxa 4950(3): 528–546. 10.11646/zootaxa.4950.3.6 33903429

[B13] Hu SQ Zhao EM (1966) Three new species of reptiles from Szechwan. Acta Zootaxon Sinica 3(2): 158–164. [In Chinese with English abstract]

[B14] Hu SQ Zhao EM Liu CZ (1973) A survey of amphibians and reptiles in Kweichow Province, including a herpetological analysis. Dong Wu Xue Bao 19(2): 149–181. [In Chinese with English abstract]

[B15] Huang RY Peng LF Yu L Huang TQ Jiang K Ding L Chang JK Yang DC Xu YH Huang S (2021) A new species of the genus *Achalinus* from Huangshan, Anhui, China (Squamata: Xenodermidae). Asian Herpetological Research 12(2): 178–187. 10.16373/j.cnki.ahr.200075

[B16] Kalyaanamoorthy S Minh BQ Wong TKF Haeseler AV Jermiin LS (2017) ModelFinder: Fast model selection for accurate phylogenetic estimates. Nature Methods 14(6): 587–589. 10.1038/nmeth.4285 28481363 PMC5453245

[B17] Koshikawa A (1982) Three new species of reptiles from Hainan Island, Guangdong Province. Smithsonian Herpetological Information Service 53(53): 1–10. 10.5479/si.23317515.53.1

[B18] Li ZS Mo JW Gu YL Liu S Fei DB Yang DD (2010) Investigation and analysis on amphibian and reptile resources in Nanyue Hengshan National Reserve. Hunan Forestry Science & Technology 37(1): 20–23, 29. [In Chinese with English abstract]

[B19] Li K Yu M Wu YY Liao LH Tang K Liu Q Guo P (2020) A new species of the genus *Achalinus* (Squamata: Xenodermatidae) from southeastern Yunnan Province, China. Zootaxa 4860(1): 116–128. 10.11646/zootaxa.4860.1.6 33056175

[B20] Li K Wu YY Xu RY Zhu F Ren JL Guo P Dong BJ (2021) A new species of the *Achalinusrufescens* complex (Xenodermidae: *Achalinus*) from Fujian Province, China. Zootaxa 5026(2): 239–254. 10.11646/zootaxa.5026.2.5 34810931

[B21] Li H Zhu LQ Zhang XT Xiao HG Peng DY Zhang ZQ Mo XY (2023) A new snake record in Hunan Province: *Achalinusyunkaiensis*. Sichuan Journal of Zoology 42(4): 428–429. [In Chinese]

[B22] Luu VQ Ziegler T Van HN Van LO Hoang TT Ngo HT Le MD Tran DH Nguyen TQ (2020) A new species of *Achalinus* (Squamata: Xenodermidae) from Trang An Landscape Complex, Ninh Binh Province, Vietnam. Zootaxa 4877(1): 174–184. 10.11646/zootaxa.4877.1.8 33311331

[B23] Ma S Shi SC Jiang JP (2023a) *Achalinusyunkaiensis*, a new provincial record of Hunan Province, China, with description of an additional topotype of *A.rufescens.* Dongwuxue Zazhi. [In Chinese with English abstract]

[B24] Ma S Shi SC Xiang SJ Shu F Jiang JP (2023b) A new species of *Achalinus* Peters, 1869 (Squamata, Xenodermidae) from Hunan Province, China. ZooKeys 1166: 315–331. 10.3897/zookeys.1166.103055 PMC10848825 38328667

[B25] Meyer CP Geller JB Paulay G (2005) Fine scale endemism on coral reefs: Archipelagic differentiation in turbinid gastropods. Evolution; International Journal of Organic Evolution 59(1): 113–125. 10.1111/j.0014-3820.2005.tb00899.x 15792232

[B26] Miller AH Davis HR Luong AM Do QH Pham CT Ziegler T Lee JL Queiroz KD Reynolds RG Nguyen TQ (2020) Discovery of a new species of enigmatic odd-scaled snake (Serpentes: Xenodermidae: *Achalinus*) from Ha Giang Province, Vietnam. Copeia 108(4): 796–808. 10.1643/CH2020060

[B27] Nguyen LT Schmidt HA Haeseler AV Minh BQ (2015) IQ-TREE: A fast and effective stochastic algorithm for estimating maximum likelihood phylogenies. Molecular Biology and Evolution 32(1): 268–274. 10.1093/molbev/msu300 25371430 PMC4271533

[B28] Ota H Toyama M (1989) Taxonomic re-definition of *Achalinusformosanus* Boulenger, 1908 (Xenoderminae: Colubridae: Ophidia), with description of a new subspecies. Copeia 1989(3): 597–602. 10.2307/1445485

[B29] Pham AV Pham CT Le MD Ngo HT Ong AV Ziegler T Nguyen TQ (2023) *Achalinusquangi*, a new odd-scaled snake species from Vietnam. Zootaxa 5270(1): 48–66. 10.11646/zootaxa.5270.1.2 37518178

[B30] Ronquist F Teslenko M Van DMP Ayres DL Darling A Hohna S (2012) MrBayes 3.2: Efficient Bayesian phylogenetic inference and model choice across a large model space. Systematic Biology 61(3): 539–542. 10.1093/sysbio/sys029 22357727 PMC3329765

[B31] Stephane G Jean-franc OD Vincent L Maria A Wim H And OG (2010) New algorithms and methods to estimate Maximum-likelihood phylogenies: Assessing the performance of PhyML 3.0. Systematic Biology 59(3): 307–321. 10.1093/sysbio/syq010 20525638

[B32] Tamura K Stecher G Kumar S (2021) MEGA11: Molecular evolutionary genetics analysis version 11. Molecular Biology and Evolution 38(7): 3022–3027. 10.1093/molbev/msab120 33892491 PMC8233496

[B33] Teynié A David P Lottier A Le MD Vidal N Nguyen TQ (2015) A new genus and species of xenodermatid snake (Squamata: Caenophidia: Xenodermatidae) from northern Lao People’s Democratic Republic. Zootaxa 3926(4): 523–540. 10.11646/zootaxa.3926.4.4 25781800

[B34] Wang J Li Y Zeng ZC Lyu ZT Sung YH Li YY Lin CY Wang YY (2019) A new species of the genus *Achalinus* from southwestern Guangdong Province, China (Squamata: Xenodermatidae). Zootaxa 4674(4): 471–481. 10.11646/zootaxa.4674.4.6 31715996

[B35] Xu RY Li K Zhang H He H Zhu F Wu YY Guo P (2023) Expanded description of *Achalinusyangdatongi* (Serpentes: Xenodermidae). Current Herpetology 42(1): 1–8. 10.5358/hsj.42.1

[B36] Yang DC Huang RY Jiang K Burbrink FT Gong YN Yu J Zhang Y Huang TQ Huang S (2022) A new species of the genus *Achalinus* (Squamata: Xenodermidae) from Ningshan County, Shaanxi Province, China. Zootaxa 5190(1): 127–140. 10.11646/zootaxa.5190.1.5 37045178

[B37] Yang DC Xu YH Wu JX Gong YA Huang RY Xiang J Feng ZL Huang TQ Huang S (2023) A new species of the genus *Achalinus* (Squamata: Xenodermidae) from Nanning, Guangxi, China. Zootaxa 5319(3): 389–402. 10.11646/zootaxa.5319.3.5 37518224

[B38] Yu M Li K Liu Q Yang K Wu YY Guo P (2020) First record of the *Achalinusyunkaiensis* from Maoershan National Nature Reserve, Guangxi, China. Dongwuxue Zazhi 55(6): 793–796. [In Chinese with English abstract]

[B39] Zhang D Gao FL Jakovlić I Zou H Zhang J Li WX Wang GT (2020) PhyloSuite: An integrated and scalable desktop platform for streamlined molecular sequence data management and evolutionary phylogenetics studies. Molecular Ecology Resources 20(1): 348–355. 10.1111/1755-0998.13096 31599058

[B40] Zhang CW Liu K Huang RY Hu TL Yu L Sun RL Zhang YC Wen J Zhang BW (2023) A new species of the genus *Achalinus* (Squamata: Xenodermidae) from the Dabie Mountains, Anhui, China. Animals (Basel) 13(4): 708. 10.3390/ani13040708 36830495 PMC9952718

[B41] Zhao EM (2006) Snakes of China. I. Anhui Science and Technology Publishing House, Hefei, 372 pp. [In Chinese]

[B42] Zhao EM Huang MH Zong Y (1998) Fauna Sinica: Reptilia. Vol. 3. Squamata, Serpentes. Science Press, Beijing, 522 pp. [In Chinese]

[B43] Ziegler T Nguyen TQ Pham CT Nguyen TT Pham AV Schingen VS Nguyen TT Le MD (2019) Three new species of the snake genus *Achalinus* from Vietnam (Squamata: Xenodermatidae). Zootaxa 4590(2): 249–269. 10.11646/zootaxa.4590.2.3 31716093

[B44] Zong Y Ma J (1983) A new species of the genus *Achalinopsis* from Jiangxi and the restoration of this genus. Acta Herpetologica Sinica 2(2): 61–63. [In Chinese with English abstract]

